# Characterization of 2-(2-nitro-4-trifluoromethylbenzoyl)-1,3-cyclohexanedione resistance in pyomelanogenic *Pseudomonas aeruginosa* DKN343

**DOI:** 10.1371/journal.pone.0178084

**Published:** 2017-06-01

**Authors:** Laura M. Ketelboeter, Sonia L. Bardy

**Affiliations:** Department of Biological Sciences, University of Wisconsin-Milwaukee, Milwaukee, Wisconsin, United States of America; Universite Paris-Sud, FRANCE

## Abstract

Pyomelanin is a reddish-brown pigment that provides bacteria and fungi protection from oxidative stress, and is reported to contribute to infection persistence. Production of this pigment can be inhibited by the anti-virulence agent 2-(2-nitro-4-trifluoromethylbenzoyl)-1,3-cyclohexanedione (NTBC). The *Pseudomonas aeruginosa* clinical isolate DKN343 exhibited high levels of resistance to NTBC, and the mechanism of pyomelanin production in this strain was uncharacterized. We determined that pyomelanin production in the clinical *Pseudomonas aeruginosa* isolate DKN343 was due to a loss of function in homogentisate 1,2-dioxygenase (HmgA). Several potential resistance mechanisms were investigated, and the MexAB-OprM efflux pump is required for resistance to NTBC. DKN343 has a frameshift mutation in NalC, which is a known indirect repressor of the *mexAB-oprM* operon. This frameshift mutation may contribute to the increased resistance of DKN343 to NTBC. Additional studies investigating the prevalence of resistance in pyomelanogenic microbes are necessary to determine the future applications of NTBC as an anti-virulence therapy.

## Introduction

Pyomelanin has several functions that are involved in helping bacteria and fungi survive in host organisms or the environment. First, pyomelanin can provide protection from oxidative stress and UV light [1–8]. Additionally, pyomelanin is involved in electron transfer, iron reduction, and iron acquisition [9–12]. There are conflicting studies on the role of pyomelanin in the infection process. The majority of reports show pyomelanin is involved in infection persistence [2, 13, 14], although in *Vibrio campbellii* pyomelanin production resulted in reduced virulence [15].

Pyomelanin is a negatively charged, extracellular, reddish-brown pigment that results from a defect in the tyrosine catabolism pathway. In a functional tyrosine catabolism pathway, 4-hydroxyphenylpyruvate is converted into homogentisate (HGA) by 4-hydroxyphenylpyruvate dioxygenase (Hpd) (S1 Fig). HGA is then converted into 4-maleylacetoacetate by homogentisate 1,2-dioxygenase (HmgA). Mutations or chromosomal deletions that result in loss of HmgA function lead to an accumulation of HGA [1, 2, 16, 17]. HGA is secreted from the cell via the HatABCDE ABC transporter, auto-oxidizes, and self-polymerizes to form pyomelanin [2, 18–20]. A reduction in enzyme activity in the latter part of the tyrosine catabolism pathway (HmgA, MaiA, and FahA) relative to the upper part of the pathway can also lead to pyomelanin production [21].

Many clinical and environmental bacterial species have been reported to produce pyomelanin, including *Pseudomonas aeruginosa*, *Burkholderia cepacia* complex, *Vibrio cholerae*, *Legionella pneumophila*, *Shewanella algae*, *Bacillus anthracis*, *Aeromonas media*, *Acinetobacter baumannii*, *Sinorhizobium meliloti*, *Streptomyces coelicolor*, and *Alteromonas stellipolaris*, [4, 7, 10, 12, 22–29]. As bacteria are increasingly developing resistance to antibiotics, investigation into new therapeutic agents is necessary [30]. Because pyomelanin has several functions that would be advantageous for bacterial survival in a host and has been reported to contribute to infection persistence, compounds that inhibit pyomelanin production are being investigated as possible therapeutic agents that could be used alone or in combination with existing antimicrobials [1, 31]. We have previously shown that the Hpd inhibiting compound 2-(2-nitro-4-trifluoromethylbenzoyl)-1,3-cyclohexanedione (NTBC) reduces pyomelanin production in *P*. *aeruginosa* clinical isolates and increases sensitivity to oxidative stress [1]. NTBC binds to the ferrous form of Hpd with high affinity in what has been proposed to be an irreversible reaction [32]. While this triketone was originally developed as an herbicide, it also inhibits Hpd in humans and has been employed therapeutically to treat type 1 tyrosinemia and alkaptonuria [33].

*P*. *aeruginosa* is a Gram-negative bacterium that is able to survive in a variety of different ecological environments including soil, water, plants, and animals [34]. This organism is also an opportunistic pathogen that frequently colonizes the lungs of cystic fibrosis (CF) patients, where it forms biofilms resulting in chronic infections [35]. Additionally, it causes acute infections in burn patients [36, 37]. *P*. *aeruginosa* is capable of producing a variety of virulence factors including secreted proteins, toxins, and pigments that contribute to the infection process [38].

High levels of intrinsic and acquired antimicrobial resistance are also characteristic of *P*. *aeruginosa* and occur through a variety of mechanisms. These include outer membrane impermeability, chromosomally encoded β-lactamases, increased resistance during biofilm formation, and the presence of multiple multi-drug efflux pumps [34, 39–41]. Twelve different resistance nodulation division (RND) efflux pumps have been identified in *P*. *aeruginosa*, and four of those are known to contribute to antibiotic resistance (MexAB-OprM, MexCD-OprJ, MexEF-OprN, and MexXY-OprM) [34]. The MexAB-OprM multi-drug efflux pump is constitutively expressed, and hyperexpression of this efflux pump has been observed in multi-drug resistant clinical isolates of *P*. *aeruginosa* [40]. Hyperexpression can result from the mutagenesis of the transcriptional regulators MexR, NalC, or NalD [42–44]. Additionally the *mexAB-oprM* operon can be derepressed in response to environmental cues such as oxidative stress (MexR) or environmental contaminants [45]. MexAB-OprM has broad substrate specificity, which is demonstrated by its ability to extrude several different classes of antibiotics, as well as dyes, detergents, organic solvents, fatty acid synthesis inhibitors, and homoserine lactone [46]. Ultimately, the multitude of antimicrobial resistance mechanisms in *P*. *aeruginosa* make it difficult to treat infections.

In this study, we determined that the pyomelanogenic *P*. *aeruginosa* isolate DKN343 has a high level of resistance to NTBC. This strain was previously reported to produce pyomelanin through a HmgA-independent mechanism, [20] although that is not supported by our study. Sequencing of *hmgA* identified two point mutations, one of which alters the predicted iron cofactor binding site and results in pyomelanin production. While NTBC has previously been shown to inhibit pyomelanin production in *P*. *aeruginosa*, its effect is limited in DKN343 due to the MexAB-OprM efflux pump.

## Materials and methods

### Strains, plasmids, and growth conditions

A list of strains and plasmids is found in Table 1. Strains were grown on LB agar or in LB broth supplemented with gentamicin, tetracycline, or chloramphenicol where appropriate. Antibiotic concentrations were as follows: gentamicin 10 μg ml^-1^ (*E*. *coli*), 50 μg ml^-1^ (PAO1 *hpd*::*tn*, PAO1 *hmgA*::*tn*, and DKN343 in broth media), and 100 μg ml^-1^ (DKN343 on agar); tetracycline 10 μg ml^-1^ (*E*. *coli*) and 195 μg ml^-1^ (DKN343); chloramphenicol 5 μg ml^-1^. Strains were grown at 37°C unless otherwise indicated.

**Table 1:** Strains and plasmids used in this study.

Strains	Description	Source
*P*. *aeruginosa* strains		
PAO1	Wild type (Iglewski strain)	Carrie Harwood
PAO1 *hpd*::*tn* (PW2577)	*hpd*-H02::ISlacZ/hah	University of Washington
PAO1 *hmgA*::*tn* (PW4489)	*hmgA*-C03::ISphoA/hah	University of Washington
PAO1 *hmgA*::*tn*Δ*hpd*	In-frame deletion of *hpd* in *hmgA*::*tn*	This study
PAO1 *hmgA*::*tn*Δ*mexA*	In-frame deletion of *mexA* in *hmgA*::*tn*	This study
PAO1 *hmgA*::*tn*ΔPA0242	In-frame deletion of PA0242 in *hmgA*::*tn*	This study
DKN343	Clinical isolate of *P*. *aeruginosa* from sputum sample, pyomelanin producer	[20]
DKN343Δ*hpd*	In-frame deletion of *hpd* in DKN343	This study
DKN343Δ*mexA*	In-frame deletion of *mexA* in DKN343	This study
DKN343ΔPA14_03000	In-frame deletion of PA14_03000 in DKN343	This study
*E*. *coli* strains		
DH5α	*fhuA2 Δ(argF-lacZ)U169 phoA glnV44 Φ80 Δ(lacZ)M15 gyrA96 recA1 relA1 endA1 thi-1 hsdR17*	New England Biolabs
S17-1	Tp^R^ Sm^R^ *recA thi pro hsdR*^-^ M^+^ RP4 2-Tc::Mu-Km::Tn7 λpir	[47]
**Plasmids**	**Description**	**Source**
pEX18Tc	Suicide vector for making deletion mutants, Tc^R^	[48]
pEX19Gm	Suicide vector for making deletion mutants, Gm^R^	[48]
pEX18Tc-Δ*hpd*	pEX18Tc based plasmid for deletion of *hpd*	This study
pEX19Gm-Δ*hpd*	pEX19Gm based plasmid for deletion of *hpd*	This study
pEX18Tc-Δ*mexA*	pEX18Tc based plasmid for deletion of *mexA*	This study
pEX19Gm-Δ*mexA*	pEX19Gm based plasmid for deletion of *mexA*	This study
pEX18Tc-ΔPA0242	pEX18Tc based plasmid for deletion of PA0242	This study
pEX19Gm-ΔPA0242	pEX19Gm based plasmid for deletion of PA0242	This study
pSB109	Derivative of pJN105 with an enhanced ribosome binding site, 6x-His tag, Gm^R^	This study
pSB109-*hmgA*_PAO1_	*hmgA* from PAO1 in NdeI-SacI sites of pSB109	This study
pSB109-*hmgA*_A306T_	*hmgA* from PAO1 containing A306T mutation in NdeI-SacI sites of pSB109	This study
pSB109-*hmgA*_H330Y_	*hmgA* from PAO1 containing H330Y in NdeI-SacI sites of pSB109	This study
pSB109-*hmgA*_343_	*hmgA* from DKN343 in NdeI-SacI sites of pSB109	This study
pSB109-*hmgA*_T306A_	*hmgA* from DKN343 containing T306A reversion mutation in NdeI-SacI sites of pSB109	This study
pSB109-*hmgA*_Y330H_	*hmgA* from DKN343 containing Y330H reversion mutation in NdeI-SacI sites of pSB109	This study
pSB109-*mexA*	*mexA* in NdeI—SacI sites pSB109	This study
pSB109-PA0242	PA0242 in EcoRI and SacI sites of pSB109	This study

### Deletion and complementation studies

In-frame deletion mutants of *hpd*, *mexA* and PA0242 in *P*. *aeruginosa* were constructed by splicing by overlap extension (SOE) PCR with PAO1 DNA as template. Primers are listed in S1 Table. The in-frame fusions were sequenced to ensure no mutations were introduced. The deletion alleles were cloned into pEX19Gm or pEX18Tc and transformed into *E*. *coli* S17-1 for mating with *P*. *aeruginosa* PAO1 *hmgA*::*tn* and DKN343, respectively. These constructs were introduced into *P*. *aeruginosa* by conjugation, and merodiploids were selected on chloramphenicol and tetracycline or gentamicin as appropriate with overnight (PAO1 *hmgA*::*tn*) or two days (DKN343) incubation. Resolution of the merodiploids was achieved through 10% sucrose counter selection. Following screening on tetracycline or gentamicin and sucrose, the deletions were confirmed by PCR or Southern blot.

Plasmids for complementation were generated by amplifying *hmgA*, *mexA*, and PA0242 from PAO1 or DKN343 using the appropriate primers (S1 Table). The resulting PCR products were sequenced and cloned into pSB109. Single point mutations of A306T and H330Y in *hmgA*_PAO1_ were generated by site directed mutagenesis based on the QuikChange mutagenesis kit. Reversion mutations T306A and Y330H in *hmgA*_343_ were also generated by site directed mutagenesis. PCR was performed with the appropriate mutagenic primers (S1 Table) using the appropriate pSB109-*hmgA* plasmid as template. The codon change was confirmed through sequencing.

In complementation studies with His-HmgA and the His-HmgA point mutants, test tubes containing LB and gentamicin were inoculated with washed overnight cultures to a concentration of OD_600_ 0.05 (PAO1 *hpd*::*tn* and PAO1 *hmgA*::*tn*) or 0.1 (DKN343) and grown to OD_600_ 0.2–0.3 before induction with arabinose (0–0.1%) for 1.5 h. In complementation studies with His-MexA, test tubes containing LB, gentamicin, and 0.05% arabinose were inoculated with washed overnight cultures to a concentration of OD_600_ 0.05 and incubated for 24 h. Whole cell lysates were separated by SDS-PAGE. All complementation cultures were incubated for a total of 24 hours prior to being photographed.

### NTBC titrations

NTBC titrations were performed as previously described [49] with gentamicin and arabinose supplementation where appropriate. Cultures were incubated for approximately 24 hours before photos were taken.

### Sequencing of *hmgA*, *hpd*, and *mexAB-oprM* repressors from DKN343

*hmgA* and *hpd* from DKN343 were PCR amplified (for primers see S1 Table), purified, and sequenced. Three known repressors (*nalC*, *nalD*, and *mexR*) of *mexAB-oprM* were also sequenced, along with 500 bp of upstream sequence. The sequencing results were compared to *P*. *aeruginosa* PA14 to identify any mutations.

### SDS-PAGE and western blots

Whole cell lysates were analyzed by SDS-PAGE, with loading based on OD_600_. Protein samples were separated on 10% gels and transferred to PVDF. Membranes were probed with mouse-α-His antibody (1:3000) followed by peroxidase conjugated sheep-α-mouse antibody (1:10000). Signal was detected using SuperSignal West Femto Maximum Sensitivity Substrate and a CCD camera with Fotodyne software.

### Nitrocefin hydrolysis assay

The nitrocefin hydrolysis assay to determine outer membrane permeability was modified from previously described procedures [50, 51]. Overnight cultures were diluted 1:60 in 30 ml LB and incubated for 2 h at 37°C with shaking. Imipenem (0.25 μg ml^-1^) was added for 3 h to induce β-lactamase expression before cells were harvested by centrifugation (5000 xg, 10 min). Cell concentration was normalized based on OD_600_, and cell pellets were washed and concentrated in 50 mM sodium phosphate buffer (pH 7.2). Aliquots of cells were treated with 0 and 0.1 mM EDTA for 5 min. Samples were centrifuged at 15000 xg for 30 min at room temperature and β-lactamase containing supernatant was saved. β-lactamase containing supernatant was diluted in 50 mM sodium phosphate buffer (pH 7.2) and nitrocefin (Calbiochem) was added at a final concentration of 100 μM in a total volume of 1 ml. Nitrocefin hydrolysis was measured spectrophotometrically at 482 nm and β-lactamase activity (U/L) was calculated using the extinction coefficient of 17.4 mM^-1^ cm^-1^ for nitrocefin. The enzyme activity per OD_600_ of cells was calculated and averaged over five biological replicates. Statistical analysis was performed by ANOVA, followed by Tukey HSD post-hoc analysis using R (version 3.2.4).

### Photo analysis and image manipulation

All photos were taken on a Canon PowerShot A480 digital camera. Photos were cropped in Adobe Photoshop and image resolution was adjusted to 600 pixels/inch.

## Results

**Fig 1:**
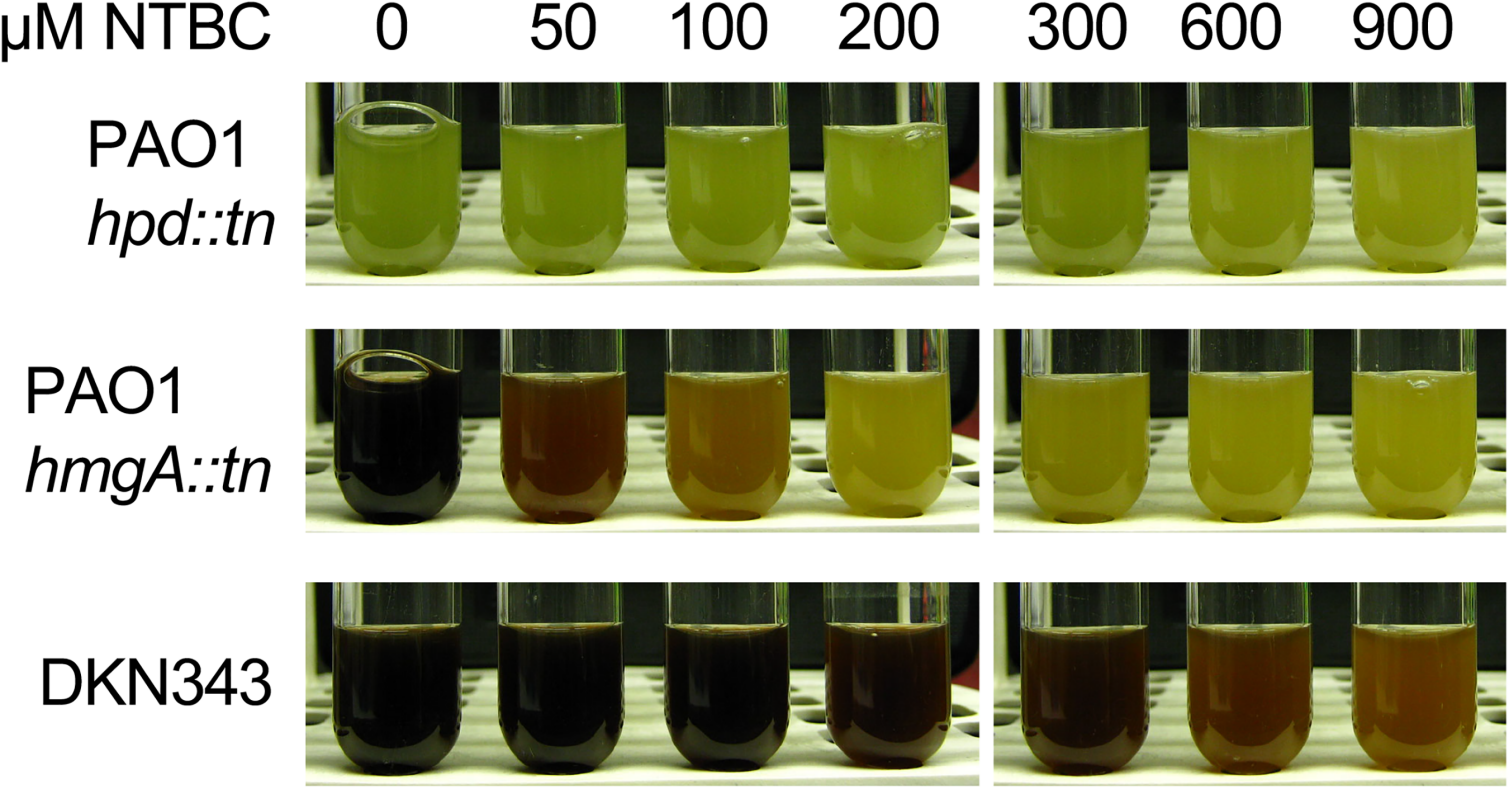
NTBC treatment reduced pigment production in pyomelanogenic *P*. *aeruginosa*. Strains were grown in LB with the indicated concentrations of NTBC. The non-pyomelanogenic control PAO1 *hpd*::*tn* showed no pigment change in response to NTBC treatment. The pyomelanin producers PAO1 *hmgA*::*tn* and DKN343 showed dose dependent reductions in pigmentation with increasing concentrations of NTBC. PAO1 *hmgA*::*tn* was the positive control for pyomelanin production. The clinical isolate DKN343 was less affected by NTBC than the laboratory strain PAO1 *hmgA*::*tn*, as indicated by the higher concentrations of NTBC required to reduce pyomelanin production.

Previous studies in our laboratory have focused on the ability of NTBC to inhibit pyomelanin production in clinical isolates of *P*. *aeruginosa* [1]. In the course of this work, we tested the efficacy of NTBC to inhibit pyomelanin production in a number of clinical and laboratory pyomelanogenic isolates of *P*. *aeruginosa*. In these studies DKN343 revealed a high level of resistance to NTBC. The ability of DKN343 to produce pyomelanin when in the presence of high concentrations of NTBC (900 μM) suggests that NTBC is unable to inhibit Hpd activity (Fig 1). DKN343 was isolated from a sputum sample (D. Newman, personal communication) and was reported to have wild type *P*. *aeruginosa* PA14 sequence in *hmgA* [20]. Pyomelanin production typically results from either point mutations or deletions of *hmgA*, or an imbalance in enzyme activity within the tyrosine catabolism pathway [2, 16, 21]. We therefore investigated the mechanism of pyomelanin production and NTBC resistance in this clinical isolate.

### Pyomelanin production in DKN343 results from a loss of function mutation in HmgA

**Fig 2:**
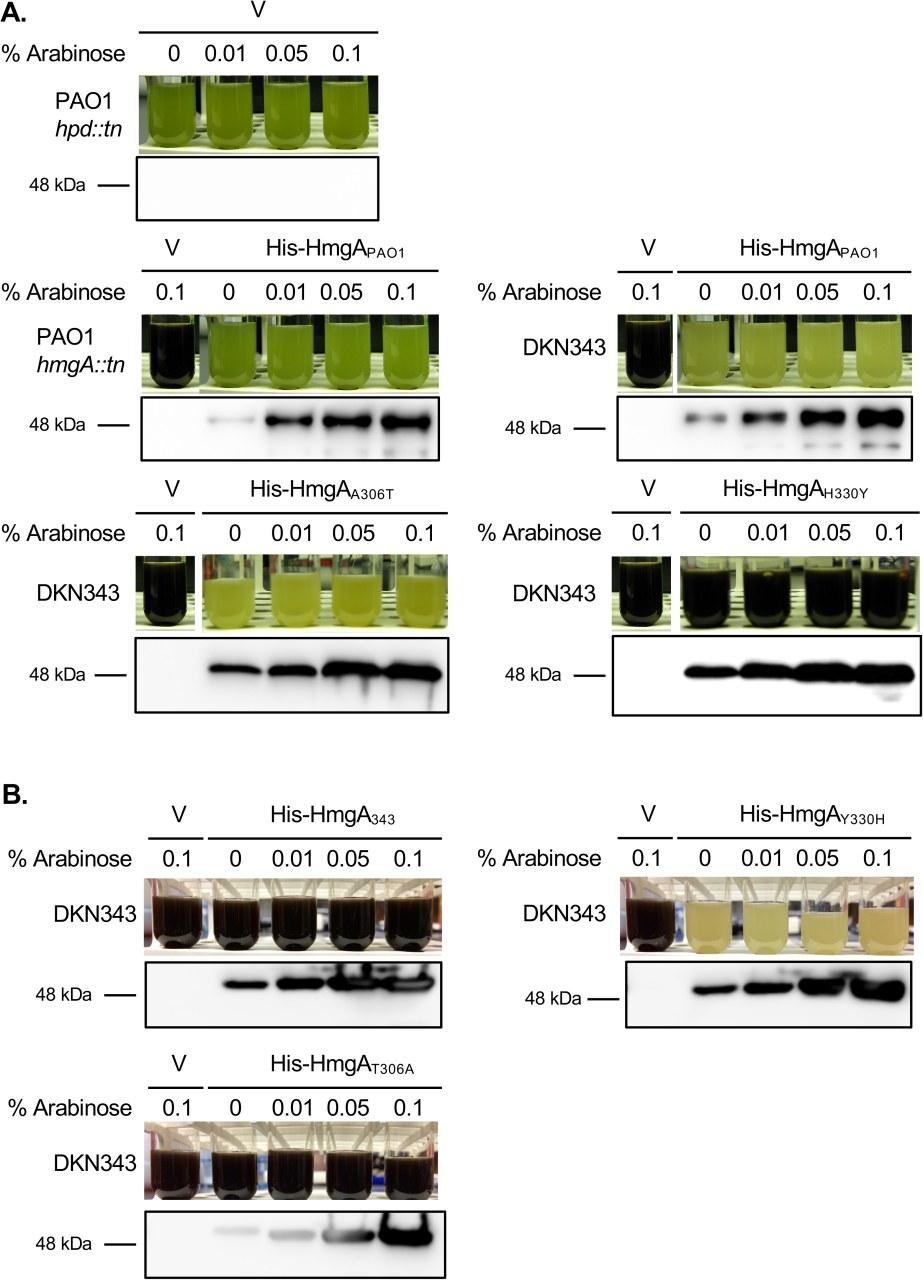
Pyomelanin production in DKN343 results from a loss of function mutation in HmgA. The H330Y mutation in HmgA resulted in pyomelanin production in DKN343. (A) Expression of His-HmgA from PAO1 *in trans* eliminated pyomelanin production in DKN343, which demonstrated that a defect in HmgA function is responsible for pyomelanin production in this strain. HmgA_H330Y_ did not alleviate pyomelanin production in DKN343, despite similar levels of expression as the wild type protein, indicating that the H330 residue is important for PAO1 HmgA function. HmgA_A306T_ was functional, as indicated by the absence of pyomelanin production in DKN343. Anti-His western blots for HmgA_PAO1_ and mutated versions of His-HmgA showed increased protein expression with increasing concentrations of arabinose. PAO1 *hpd*::*tn* was the non-pyomelanogenic control, while PAO1 *hmgA*::*tn* functioned as a positive control for pyomelanin production resulting from a defect in the tyrosine catabolism pathway. (B) Overexpression of His-HmgA_343_ did not alleviate pyomelanin production in DKN343. Introduction of the Y330H reversion mutation restored HmgA_343_ function and alleviated pyomelanin production in DKN343. The reversion mutant HmgA_T306A_ was non-functional in DKN343 and pyomelanin production was retained. Anti-His western blots for HmgA_343_ and both reversion mutants showed increased protein expression with increasing concentrations of arabinose.

Because NTBC binding to Hpd inhibits the synthesis of HGA [32], the ability of NTBC to partially inhibit pyomelanin production in DKN343 suggested that this pigmentation resulted from defects in the tyrosine catabolism pathway. Although *hmgA*_DKN343_ had previously been sequenced and found to correspond to wild type PA14 *hmgA* [20], we identified two mutations in HmgA_DKN343_ when compared to HmgA_PA14_; A306T and H330Y (S2 Fig). Importantly, the H330Y mutation occurred in the HmgA iron cofactor binding site, which is expected to be required for functional HmgA. To determine if these mutations contribute to pyomelanin production through a loss of HmgA_DKN343_ function, wild type *hmgA*_PAO1_ was expressed from an arabinose inducible plasmid in DKN343 and the control PAO1 *hmgA*::*tn* (Fig 2A). Low levels of HmgA_PAO1_ expression alleviated pyomelanin production in DKN343, demonstrating that pigment production does result from a defect in HmgA function. HmgA_PAO1_ is 99% identical to HmgA_PA14,_ with only two mismatches. These mismatches occur at amino acids 226 and 303; our data shows that these mismatches between PAO1 and PA14 do not affect the function of HmgA.

To ascertain whether the A306T or the H330Y mutation identified in DKN343 was responsible for the loss of function, site directed mutagenesis was used to introduce each mutation in *hmgA*_PAO1_, and these constructs were tested for their ability to complement the DKN343 strain (Fig 2A). Expression of PAO1 HmgA_A306T_ eliminated pigmentation, demonstrating that this mutation does not affect enzymatic activity. PAO1 HmgA_H330Y_ did not alleviate pyomelanin production, which indicated the functional importance of the H330Y mutation. Reversion mutations resulting in T306A and Y330H amino acid substitutions were individually introduced into *hmgA* from DKN343. These constructs were expressed from an arabinose inducible plasmid in DKN343 to determine which mutation was responsible for pyomelanin production. As indicated by a loss of pyomelanin production, function was only restored to HmgA_343_ when the histidine residue was present at the iron cofactor binding site (H330). Western blots revealed stable protein expression with all versions of HmgA, thereby eliminating the possibility of insufficient protein expression as the cause of pyomelanin production.

These data clearly indicate that pyomelanin production in DKN343 occurs in the conventional manner: A loss of function mutation in HmgA prevents the conversion of HGA to 4-maleylacetoacetate. The intracellular HGA is then secreted from DKN343 by the HatABCDE ABC transporter before it is auto-oxidized and self-polymerized into pyomelanin [20].

### Resistance to NTBC is mediated through the MexAB-OprM multi-drug efflux pump

Studies on the NTBC-mediated inhibition of *Streptomyces avermitilis* 4-hydroxyphenylpyruvate dioxygenase (HppD) revealed that NTBC binds irreversibly to the ferrous metal center in the active site of the enzyme [32]. In previous studies of pyomelanogenic *P*. *aeruginosa*, NTBC treatment inhibited pyomelanin production in a dose dependent manner [1]. Early assays on DKN343 revealed 900 μM NTBC only partially inhibited pyomelanin production (Fig 1). This is a notable increase (approximately 12 fold) in the amount of NTBC required compared to the pyomelanogenic laboratory strain PAO1 *hmgA*::*tn*, and suggested this clinical isolate possessed pre-existing resistance to NTBC. Because NTBC has been proposed as a potential therapeutic agent for pyomelanogenic infections, it was necessary to ascertain the mechanism of resistance. We investigated several ways DKN343 could remain unaffected by NTBC including target alterations, sequestration of NBTC, impermeability of the outer membrane, and efflux via MexAB-OprM.

A common mechanism of antibiotic resistance in bacteria is through target alterations. We therefore investigated the possibility that the high level of resistance in DKN343 was due to the inability of NTBC to inactivate Hpd_DKN343_. Analysis of the *hpd*_DKN343_ sequence failed to identify any mutations relative to the PA14 *hpd* sequence, suggesting that NTBC should be able to bind and inactivate Hpd_DKN343_. As the difference in NTBC sensitivity may be strain dependent (PAO1 vs PA14), we also compared the identical PA14/DKN343 sequence of Hpd with PAO1 (S3 Fig). There is one amino acid difference; an alanine is found at location 263 in PA14, and in PAO1 an aspartate is found at the similar location. It is unlikely that this difference is the cause of resistance to NTBC, as an acidic residue (glutamic acid) is found in the same location in the pig and human HppD [52]. Additionally, co-crystallization studies of NTBC with HppD (*Streptomyces avermitilis*) demonstrates interactions with His187, His270 and Glu349 with the phenyl ring of NTBC sandwiched between Phe336 and Phe364 [52]. These amino acids are all conserved in Hpd of *P*. *aeruginosa* PAO1 and PA14 (S3 Fig).

We also examined the *P*. *aeruginosa* genomes for proteins with homology to the active site of Hpd with the possibility that these proteins are binding and sequestering NTBC, thereby increasing the concentration needed for inhibition of Hpd and pyomelanin production. Using BlastP, we identified the hypothetical protein PA0242/PA14_03000, wherein the C-terminal region of the protein has homology to Hpd, including conservation surrounding the iron cofactor binding sites where NTBC binds (S4A Fig). Deletion of PA0242 in PAO1 *hmgA*::*tn* and PA14_03000 in DKN343 did not increase sensitivity of these strains to NTBC (S4B Fig), demonstrating that these proteins do not contribute to resistance against NTBC.

*P*. *aeruginosa* is known to have low outer membrane permeability, which can contribute to antimicrobial resistance [34]. We therefore investigated if increasing outer membrane permeability increased sensitivity to NTBC. Treatment with 0.1 mM EDTA was sufficient to increase outer membrane permeability in PAO1 *hmgA*::*tn* and DKN343 when compared to the untreated strain. Nitrocefin hydrolysis by β-lactamase released from the periplasm was used as an indicator of outer membrane permeability (S5A Fig). Treatment of pyomelanogenic strains with 0.1 mM EDTA and sub-inhibitory concentrations of NTBC (50 μM for PAO1 *hmgA*::*tn* and 900 μM for DKN343) revealed that increasing outer membrane permeability did not increase sensitivity to NTBC (S5B Fig). In both PAO1 *hmgA*::*tn* and DKN343, the relative amount of pyomelanin produced in the NTBC treated strains was very similar regardless of EDTA treatment. These results demonstrate that outer membrane impermeability does not contribute to the heightened NTBC resistance in DKN343. The non-pyomelanogenic control PAO1 *hpd*::*tn* had a similar increase in outer membrane permeability with EDTA treatment, and growth of all strains was unaffected by EDTA (data not shown).

**Fig 3:**
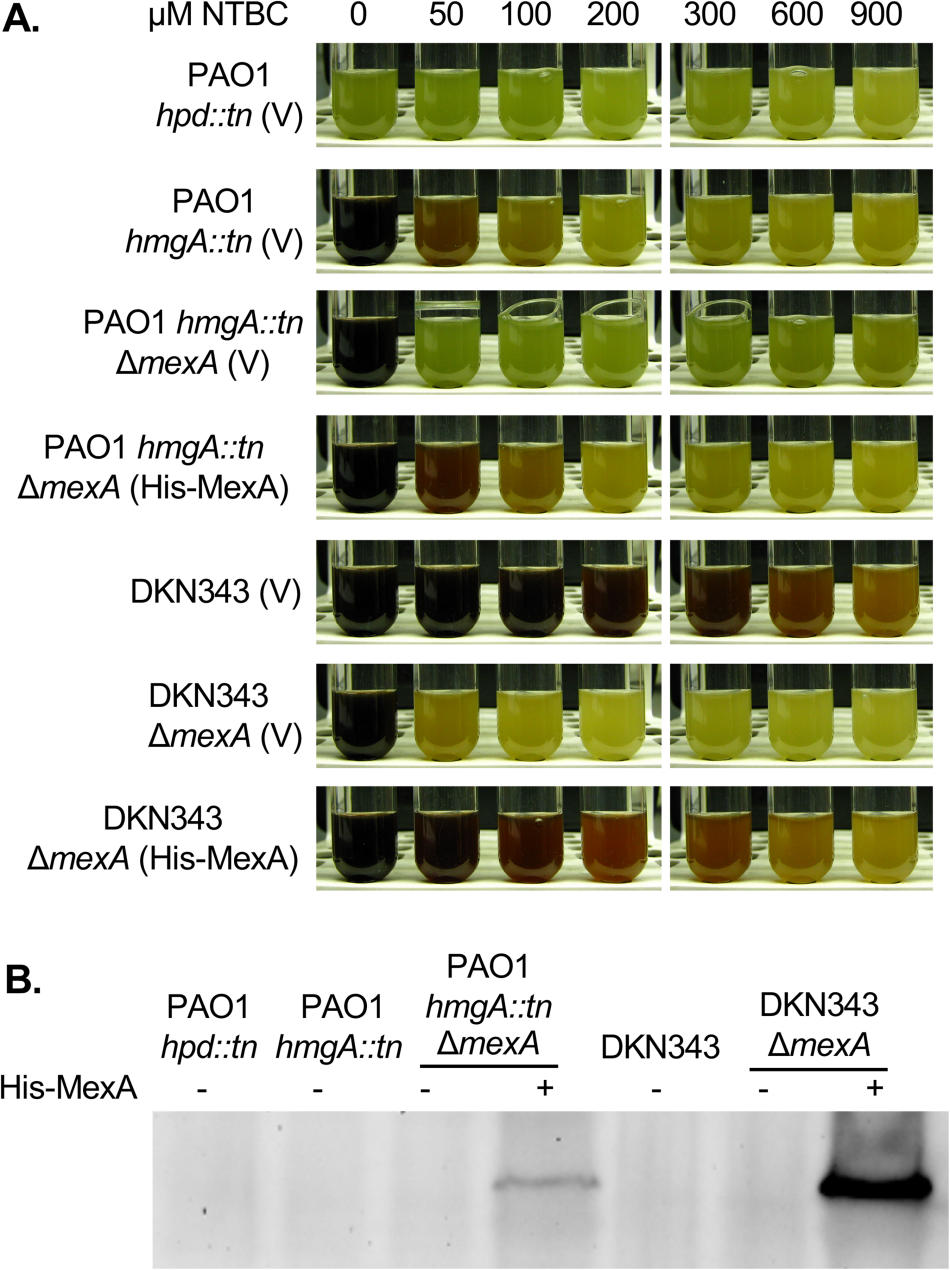
Resistance to NTBC is mediated through the MexAB-OprM multidrug efflux pump. (A) Deletion of *mexA* in PAO1 *hmgA*::*tn* and DKN343 increased sensitivity to NTBC as indicated by reduced levels of pyomelanin. Strains complemented with His-MexA showed restoration of pyomelanin production in the presence of NTBC. PAO1 *hpd*::*tn* (V) was the pyomelanin non-producer control. Cultures were grown in LB with gentamicin and 0.05% arabinose with the indicated concentrations of NTBC. (B) The relative levels of His-MexA was determined through western blotting of whole cell lysates from PAO1 *hmgA*::*tn*Δ*mexA* and DKN343Δ*mexA*. MexA was expressed at higher levels in in DKN343Δ*mexA* compared to PAO1 *hmgA*::*tn*Δ*mexA*. Cultures were grown for 24 hours in LB with gentamicin and 0.05% arabinose.

Multi-drug efflux pumps contribute to antimicrobial resistance by extruding a broad range of compounds from the bacterial cell [41]. We therefore tested the contribution of the constitutively active multi-drug efflux pump MexAB-OprM to NTBC resistance. Increased sensitivity to NTBC was observed in the *mexA* deletion mutants in both PAO1 *hmgA*::*tn* and DKN343, as indicated by reduced levels of NTBC that are sufficient to inhibit pyomelanin production (50 μM for PAO1 *hmgA*::*tn* and 100 μM for DKN343). *mexA* encodes the membrane fusion protein of this tripartite pump. Expression of His-MexA restored resistance to NTBC in PAO1 *hmgA*::*tn*. Partial complementation was seen in DKN343Δ*mexA* (Fig 3A), even though expression of His-MexA was higher in DKN343Δ*mexA* compared to PAO1 *hmgA*::*tn*Δ*mexA* after 24 hours (Fig 3B). This partial complementation may be the result of the epitope-tag interfering with protein interactions. Alternatively, the stoichiometry of the efflux pump components may be altered by a high level of MexA expression, thereby interfering with pump formation. Ultimately, these results indicate that the MexAB-OprM multi-drug efflux pump mediates resistance to NTBC.

**Fig 4:**
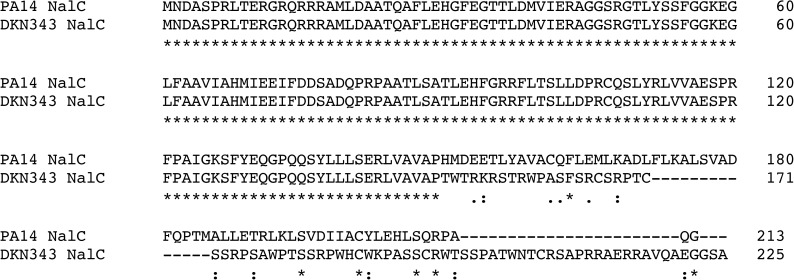
A frameshift mutation in *nalC*_DKN343_ significantly alters NalC sequence. Clustal O (1.2.0) sequence alignment of *P*. *aeruginosa* PA14 NalC (PA14_16280) and DKN343 NalC amino acid sequence. *nalC*_DKN343_ has a single base pair deletion 448 bp into the gene, resulting in a frameshift that causes a significant change in protein sequence after P149. Asterisks indicate invariant amino acids; colons indicate conservation between groups of strongly similar properties; periods indicate conservation between groups of weakly similar properties.

As our results showed that the clinical isolate DKN343 was able to produce pyomelanin in the presence of 900 μM NTBC and the tripartite efflux pump MexAB-OprM is required for this resistance, it is tempting to speculate that overexpression of the MexAB-OprM system is responsible for the increased resistance to NTBC when compared to the laboratory strain. Expression of the *mexAB-oprM* operon is transcriptionally regulated by MexR, NalC and NalD [44]. Sequencing of these regulators and their corresponding upstream regions (500 bp) revealed wild type sequence for *mexR* and *nalD*, when compared to PA14. *nalC*_DKN343_ contains a frameshift mutation that significantly alters the sequence of NalC after P149 (Fig 4.) Normally NalC regulates transcription of *mexAB-oprM* by repressing expression of the divergent PA3720-*armR* operon [43]. ArmR is an anti-repressor that functions by binding MexR and negatively impacting the ability of MexR to bind to the promoter of *mexAB-oprM*. The mutation in NalC of DKN343 may contribute to the increased resistance to NTBC.

## Discussion

Our investigations into the possible application of NTBC as a therapeutic agent against pyomelanogenic bacteria lead us to test this triketone against the clinical *P*. *aeruginosa* isolate DKN343. Because high levels of NTBC were insufficient to inhibit pyomelanin production, we examined both the mechanism of pyomelanin production and resistance to NTBC.

DKN343 produces pyomelanin as a result of a single amino acid change in HmgA that results in a loss of function. While the identification of a loss of function mutation in HmgA that results in pyomelanin production is in contrast with the previous studies on this isolate [20], it is not entirely unexpected given a number of studies have also identified mutations in *hmgA* as a cause for pyomelanin production in bacteria [25, 53]. In a *Burkholderia cenocepacia* pyomelanogenic CF clinical isolate, a single point mutation in *hmgA* resulted in an amino acid change from a glycine to an arginine at residue 378, and this mutation was conserved in three of four pigmented *B*. *cepacia* complex strains [54]. This G378R mutation in *B*. *cenocepacia* was located in the iron cofactor binding region of HmgA, which could affect iron binding and subsequent enzyme function [54]. The *B*. *cenocepacia* data are in accordance with our results, in which the H330Y mutation in HmgA_DKN343_ was located at one of the iron cofactor binding sites. The importance of this residue is clearly indicated by the failure of PAO1 HmgA_H330Y_ to alleviate pyomelanin production as well as the restoration of function through the introduction of the reversion mutation Y330H into DKN343 HmgA.

Since many bacterial and fungal pathogens produce pyomelanin, and pyomelanin has been reported to increase pathogenicity, inhibition of pyomelanin production is a potential target for a therapeutic agent. A recent study in *L*. *pneumophila* identified a compound that inhibited phenylalanine hydroxylase, which converts phenylalanine to tyrosine, and subsequently inhibited pyomelanin production [31]. This study demonstrated that targeting other enzymes in the phenylalanine/tyrosine catabolism pathway inhibits pyomelanin production in bacteria. Targeting phenylalanine hydroxylase may have limited effectiveness in the inhibition of pyomelanin production, however, as tyrosine can still be catabolized to the pyomelanin precursor HGA. Because NTBC inhibits a later step in the catabolic pathway, the ability to use both phenylalanine and tyrosine to synthesize pyomelanin is inhibited. While NTBC shows promise as a therapeutic agent against pyomelanogenic bacteria [1], its effectiveness may be limited against bacteria with a high level of pre-existing antibiotic resistance.

*P*. *aeruginosa* has high levels of intrinsic and acquired antimicrobial resistance, which can make it difficult to treat infections. Resistance to antimicrobial agents is mediated by several mechanisms, including low outer membrane permeability, several multi-drug efflux pumps, a chromosomally encoded β-lactamase, and biofilm formation [34]. This study revealed that the constitutively expressed multi-drug efflux pump MexAB-OprM was required for NTBC resistance. This was clearly indicated by the inhibition of pyomelanin production in DKN343 *mexA* mutants following treatment with only 100 μM NTBC as compared to the parent strain, where pyomelanin production was partially inhibited by 900 μM NTBC. MexAB-OprM contributes to intrinsic antimicrobial resistance in *P*. *aeruginosa* by extruding several classes of antibiotics, as well as dyes, detergents, organic solvents, and fatty acid synthesis inhibitors [46]. A number of recent studies on clinical and environmental *P*. *aeruginosa* isolates have emphasized the importance of the MexAB-OprM efflux system in antibiotic resistance [44, 55, 56].

The relative level of resistance to NTBC in DKN343 that could be attributed to the MexAB-OprM efflux pump was approximately 12-fold greater compared to the lab strain. Because this increased resistance could either be due to hyperexpression of *mexAB-oprM* or increased activity of this pump, the known transcriptional regulators *mexR*, *nalC* and *nalD* were sequenced. While *mexR* and *nalD* had sequences identical to PA14, *nalC* contained a frameshift mutation which significantly alters NalC sequence and function. It has been shown that mutations in the MexR, NalC, or NalD regulators of *mexAB*-*oprM* transcription result in hyperexpression of the operon in clinical isolates and laboratory strains of *P*. *aeruginosa* [40, 42, 43, 45, 57]. It is likely NalC_DKN343_ is non-functional, which could result in increased expression of the anti-repressor ArmR. ArmR is known to interact with MexR, thereby blocking dimer formation and the ability to bind to the to the P1 region of the *mexAB-oprM* promoter. If ArmR is overexpressed in DKN343 due to the NalC mutation, this would lead to increased expression of *mexAB-oprM* [58]. We acknowledge that there may be additional, albeit minor, factors contributing to NTBC resistance in DKN343. Even with the deletion of *mexA*, DKN343 still has a residual amount of pyomelanin produced at 50 μM NTBC, in contrast to the lab strain PAO1 *hmgA*::*tn*, which is null for pyomelanin production at this concentration of NTBC. In summary, while NTBC is able to inhibit pyomelanin production in *P*. *aeruginosa*, the constitutively active multi-drug efflux pump MexAB-OprM may limit the effectiveness of this compound against *P*. *aeruginosa*, as evidenced by DKN343. Future studies focused on the prevalence of NTBC resistance in pyomelanogenic microbes are required to determine the potential viability of this triketone as a therapeutic targeting bacterial infections.

## Supporting information

S1 Fig Tyrosine catabolism pathway in *P*. *aeruginosa*. Pyomelanin is produced via inactivation of HmgA, which results in secretion of HGA from the cell and subsequent auto-oxidation and self-polymerization to form pigment. NTBC binds to Hpd to prevent formation of HGA and pyomelanin production. PhhC, aromatic amino acid aminotransferase; Hpd, 4-hydroxyphenylpyruvate dioxygenase; HmgA, homogentisate 1,2-dioxygenase; MaiA, maleylacetoacetate isomerase; FahA, fumarylacetoacetase; HatABCDE, HatABCDE ABC transporter.
(PDF)

S2 Fig Two amino acid changes were identified in the HmgA protein sequence from the clinical isolate DKN343 compared to the HmgA sequence from *P*. *aeruginosa* PA14. The two amino acid changes, A306T and H330Y, are highlighted in red. The H330Y change occurred in the iron cofactor binding site of HmgA. Clustal O (1.2.1) multiple sequence alignment of the HmgA protein from PA14 and DKN343 was used to identify the amino acid changes in DKN343. Asterisks indicate invariant amino acids; colons indicate conservation between groups of strongly similar properties; periods indicate conservation between groups of weakly similar properties.
(PDF)

S3 Fig Sequence differences in Hpd are not involved in NTBC resistance. Clustal O (1.2.1) multiple sequence alignment of the Hpd protein from PAO1, PA14 and DKN343 was used to identify the amino acid changes in DKN343. Hpd of DKN343 and PA14 are identical, with one amino acid difference to Hpd PAO1. Asterisks indicate invariant amino acids; colons indicate conservation between groups of strongly similar properties; periods indicate conservation between groups of weakly similar properties.
(PDF)

S4 Fig PA0242/PA14_03000 is not involved in NTBC resistance. (A) Clustal O (1.2.0) sequence alignment of *P*. *aeruginosa* PAO1 and PA14 Hpd, PA0242 and PA14_03000 amino acid sequence. Hpd has sequence similarity to the C-terminal portion of the hypothetical proteins PA0242 and PA14_03000. The iron cofactor binding sites for Hpd (H168, H246, and E325) are highlighted in red. Asterisks indicate invariant amino acids; colons indicate conservation between groups of strongly similar properties; periods indicate conservation between groups of weakly similar properties. (B) Deletion of PA0242/PA14_03000 had no effect on NTBC sensitivity in the pyomelanin producers *hmgA*::*tn* and DKN343, respectively. *hpd*::*tn* was the non-pyomelanogenic control, while *hmgA*::*tn* was a positive control for pyomelanin production. Cultures were grown with the indicated concentrations of NTBC.
(PDF)

S5 Fig An increase in outer membrane permeability does not result in increased sensitivity to NTBC in pyomelanogenic *P*. *aeruginosa*. (A) EDTA treatment of cells increased outer membrane permeability, as measured by extracellular β-lactamase activity via nitrocefin hydrolysis. β-lactamase activity (U/L/OD_600_) for each untreated parent strain (0 mM EDTA) was set to 100% and percent β-lactamase activity was calculated following 0.1 mM EDTA treatment. Five biological replicates were tested. ANOVA followed by Tukey HSD post-hoc analysis: **, p<0.01; ***, p<0.001, n.s.; not significant. (B) *hmgA*::*tn* and DKN343 were treated with 0 or 0.1 mM EDTA and sub-inhibitory concentrations (0, 50, or 900 μM) of NTBC, as indicated, for 24 hours. *hmgA*::*tn* was the positive control for pyomelanin production. Three biological replicates were tested in triplicate. A representative data set is shown here.
(PDF)

S1 Table Primers used in this study. (DOCX)
