# Supplementary material for: Characterization of 2-(2-nitro-4-trifluoromethylbenzoyl)-1,3-cyclohexanedione resistance in pyomelanogenic Pseudomonas aeruginosa DKN343
Source: PLoS One. 2017 Jun 1;12(6):e0178084. doi: 10.1371/journal.pone.0178084 (PMC5453437; doi:10.1371/journal.pone.0178084)
Supplement: S1 Fig — Pyomelanin is produced via inactivation of HmgA, which results in secretion of HGA from the cell and subsequent auto-oxidation and self-polymerization to form pigment. NTBC binds to Hpd to prevent formation of HGA and pyomelanin production. PhhC, aromatic amino acid aminotransferase; Hpd, 4-hydroxyphenylpyruvate dioxygenase; HmgA, homogentisate 1,2-dioxygenase; MaiA, maleylacetoacetate isomerase; FahA, fumarylacetoacetase; HatABCDE, HatABCDE ABC transporter. (PDF) [file pone.0178084.s001.pdf]

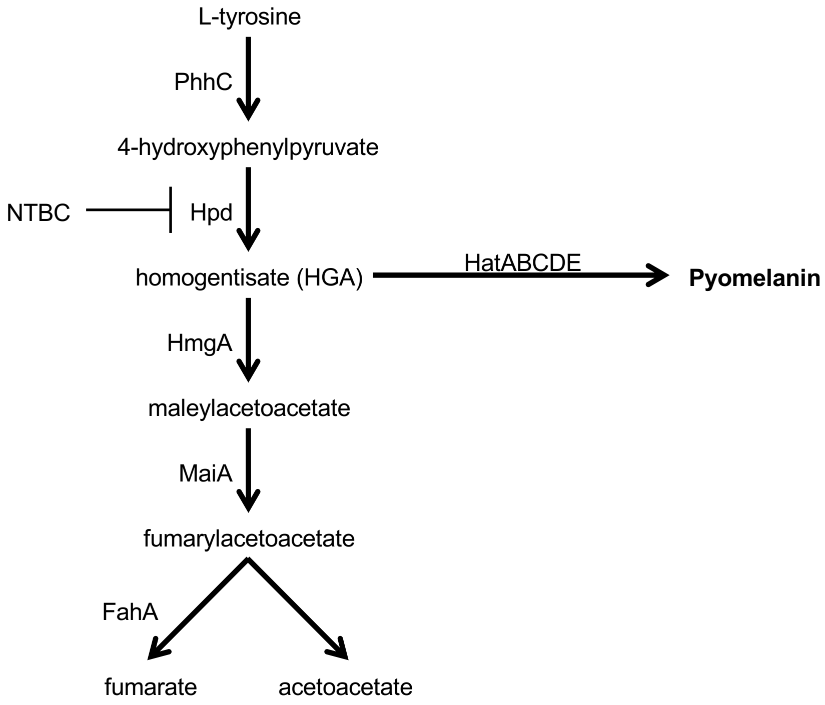

S1 Fig. Tyrosine catabolism pathway in *P. aeruginosa*. Pyomelanin is produced via inactivation of HmgA, which results in secretion of HGA from the cell and subsequent auto-oxidation and self-polymerization to form pigment. NTBC binds to Hpd to prevent formation of HGA and pyomelanin production. PhhC, aromatic amino acid aminotransferase; Hpd, 4-hydroxyphenylpyruvate dioxygenase; HmgA, homogentisate 1,2-dioxygenase; MaiA, maleylacetoacetate isomerase; FahA, fumarylacetoacetase; HatABCDE, HatABCDE ABC transporter.
