# Supplementary material for: Characterization of 2-(2-nitro-4-trifluoromethylbenzoyl)-1,3-cyclohexanedione resistance in pyomelanogenic Pseudomonas aeruginosa DKN343
Source: PLoS One. 2017 Jun 1;12(6):e0178084. doi: 10.1371/journal.pone.0178084 (PMC5453437; doi:10.1371/journal.pone.0178084)
Supplement: S2 Fig — The two amino acid changes, A306T and H330Y, are highlighted in red. The H330Y change occurred in the iron cofactor binding site of HmgA. Clustal O (1.2.1) multiple sequence alignment of the HmgA protein from PA14 and DKN343 was used to identify the amino acid changes in DKN343. Asterisks indicate invariant amino acids; colons indicate conservation between groups of strongly similar properties; periods indicate conservation between groups of weakly similar properties. (PDF) [file pone.0178084.s002.pdf]

```
PA14        MNLDSTALAYQSGFGNEFSSEALPGALPVGQNSPQKAPYGLYAELLSGTAFTMARSEARR    60
DKN343      MNLDSTALAYQSGFGNEFSSEALPGALPVGQNSPQKAPYGLYAELLSGTAFTMARSEARR    60
            ************************************************************

PA14        TWLYRITPSAKHPPFRRLERQIAGAELDAPTPNRLRWDPLALPEQPTDFLDGLLRMAANA    120
DKN343      TWLYRITPSAKHPPFRRLERQIAGAELDAPTPNRLRWDPLALPEQPTDFLDGLLRMAANA    120
            ************************************************************

PA14        PGDKPAGVSIYQYLANRSMERCFYDADGELLLVPQLGRLRLCTELGALQVEPLEIAVIPR    180
DKN343      PGDKPAGVSIYQYLANRSMERCFYDADGELLLVPQLGRLRLCTELGALQVEPLEIAVIPR    180
            ************************************************************

PA14        GMKFRVELLDGEARGYIAENHGAPLRLPDLGPIGSNGLANPRDFLAPVARYEDSRQPLQL    240
DKN343      GMKFRVELLDGEARGYIAENHGAPLRLPDLGPIGSNGLANPRDFLAPVARYEDSRQPLQL    240
            ************************************************************

PA14        VQKYLGELWACELDHSPLDVVAWHGNNVPYKYDLRRFNTIGTVSFDHPDPSIFTVLTSPT    300
DKN343      VQKYLGELWACELDHSPLDVVAWHGNNVPYKYDLRRFNTIGTVSFDHPDPSIFTVLTSPT    300
            ************************************************************

PA14        SVHGLANIDFVIFPPRWMVAENTFRPPWFHRNLMNEFMGLIQGAYDAKAGGFVPGGASLH    360
DKN343      SVHGLTNIDFVIFPPRWMVAENTFRPPWFYRNLMNEFMGLIQGAYDAKAGGFVPGGASLH    360
            *****:***********************:******************************

PA14        SCMSAHGPDAESCDKAIAADLKPHRIDQTMAFMFETSQVLRPSRAALETPALQNDYDACW    420
DKN343      SCMSAHGPDAESCDKAIAADLKPHRIDQTMAFMFETSQVLRPSRAALETPALQNDYDACW    420
            ************************************************************

PA14        ASLVSTFNPQRR      432
DKN343      ASLVSTFNPQRR      432
            ************
```

S2 Fig. Two amino acid changes were identified in the HmgA protein sequence from the clinical isolate DKN343 compared to the HmgA sequence from *P. aeruginosa* PA14. The two amino acid changes, A306T and H330Y, are highlighted in red. The H330Y change occurred in the iron cofactor binding site of HmgA. Clustal O (1.2.1) multiple sequence alignment of the HmgA protein from PA14 and DKN343 was used to identify the amino acid changes in DKN343. Asterisks indicate invariant amino acids; colon indicates conservation between groups of strongly similar properties; periods indicated conservation between groups of weakly similar properties.
