# Supplementary material for: Characterization of 2-(2-nitro-4-trifluoromethylbenzoyl)-1,3-cyclohexanedione resistance in pyomelanogenic Pseudomonas aeruginosa DKN343
Source: PLoS One. 2017 Jun 1;12(6):e0178084. doi: 10.1371/journal.pone.0178084 (PMC5453437; doi:10.1371/journal.pone.0178084)
Supplement: S3 Fig — Clustal O (1.2.1) multiple sequence alignment of the Hpd protein from PAO1, PA14 and DKN343 was used to identify the amino acid changes in DKN343. Hpd of DKN343 and PA14 are identical, with one amino acid difference to Hpd PAO1. Asterisks indicate invariant amino acids; colons indicate conservation between groups of strongly similar properties; periods indicate conservation between groups of weakly similar properties. (PDF) [file pone.0178084.s003.pdf]

```
PAO1          MNAVAKIEQHNPIGTDGFEFVEFTAPDAKGIEQLRQLFNMMGFTETAKHRSKEVFLFQQN
PA14          MNAVAKIEQHNPIGTDGFEFVEFTAPDAKGIEQLRQLFNMMGFTETAKHRSKEVFLFQQN
DKN343        MNAVAKIEQHNPIGTDGFEFVEFTAPDAKGIEQLRQLFNMMGFTETAKHRSKEVFLFQQN
              ************************************************************

PAO1          DINIVLNGSPTGHVHEFALKHGPSACAMAFRVKNASQAAAYAESQGAKLVGSHANFGELN
PA14          DINIVLNGSPTGHVHEFALKHGPSACAMAFRVKNASQAAAYAESQGAKLVGSHANFGELN
DKN343        DINIVLNGSPTGHVHEFALKHGPSACAMAFRVKNASQAAAYAESQGAKLVGSHANFGELN
              ************************************************************

PAO1          IPSLEGIGGSLLYLVDRYGDRSIYDVDFEFIEGRSANDNSVGLTYIDHLTHNVKRGQMDV
PA14          IPSLEGIGGSLLYLVDRYGDRSIYDVDFEFIEGRSANDNSVGLTYIDHLTHNVKRGQMDV
DKN343        IPSLEGIGGSLLYLVDRYGDRSIYDVDFEFIEGRSANDNSVGLTYIDHLTHNVKRGQMDV
              ************************************************************

PAO1          WSGFYERIANFREIRYFDIEGKLTGLFSRAMTAPCGKIRIPINESADDTSQIEEFIREYH
PA14          WSGFYERIANFREIRYFDIEGKLTGLFSRAMTAPCGKIRIPINESADDTSQIEEFIREYH
DKN343        WSGFYERIANFREIRYFDIEGKLTGLFSRAMTAPCGKIRIPINESADDTSQIEEFIREYH
              ************************************************************

PAO1          GEGIQHIALTTDDIYATVRKLRDNGVKFMSTPDTYYEKVDTRVAGHGEPLEQLRELNLLI
PA14          GEGIQHIALTTDDIYATVRKLRANGVKFMSTPDTYYEKVDTRVAGHGEPLEQLRELNLLI
DKN343        GEGIQHIALTTDDIYATVRKLRANGVKFMSTPDTYYEKVDTRVAGHGEPLEQLRELNLLI
              ********************** *************************************

PAO1          DGAPGDDGILLQIFTDTVIGPIFFEIIQRKGNQGFGEGNFKALFESIEEDQIRRGVI
PA14          DGAPGDDGILLQIFTDTVIGPIFFEIIQRKGNQGFGEGNFKALFESIEEDQIRRGVI
DKN343        DGAPGDDGILLQIFTDTVIGPIFFEIIQRKGNQGFGEGNFKALFESIEEDQIRRGVI
              *********************************************************
```

S3 Fig. Sequence differences in Hpd are not involved in NTBC resistance. Clustal O (1.2.1) multiple sequence alignment of the Hpd protein from PAO1, PA14 and DKN343 was used to identify the amino acid changes in DKN343. Hpd of DKN343 and PA14 are identical, with one amino acid difference to Hpd PAO1. Asterisks indicate invariant amino acids; colon indicates conservation between groups of strongly similar properties; periods indicated conservation between groups of weakly similar properties.
