# Supplementary material for: Characterization of 2-(2-nitro-4-trifluoromethylbenzoyl)-1,3-cyclohexanedione resistance in pyomelanogenic Pseudomonas aeruginosa DKN343
Source: PLoS One. 2017 Jun 1;12(6):e0178084. doi: 10.1371/journal.pone.0178084 (PMC5453437; doi:10.1371/journal.pone.0178084)
Supplement: S4 Fig — (A) Clustal O (1.2.0) sequence alignment of P. aeruginosa PAO1 and PA14 Hpd, PA0242 and PA14_03000 amino acid sequence. Hpd has sequence similarity to the C-terminal portion of the hypothetical proteins PA0242 and PA14_03000. The iron cofactor binding sites for Hpd (H168, H246, and E325) are highlighted in red. Asterisks indicate invariant amino acids; colons indicate conservation between groups of strongly similar properties; periods indicate conservation between groups of weakly similar properties. (B) Deletion of PA0242/PA14_03000 had no effect on NTBC sensitivity in the pyomelanin producers hmgA::tn and DKN343, respectively. hpd::tn was the non-pyomelanogenic control, while hmgA::tn was a positive control for pyomelanin production. Cultures were grown with the indicated concentrations of NTBC. (PDF) [file pone.0178084.s004.pdf]

A.

```
HpdPAO1         -------------------------------------MNAVAK-IEQHNPIGTDGFEFVE    22
HpdPA14         -------------------------------------MNAVAK-IEQHNPIGTDGFEFVE    22
PA0242          FNDGFRAAPTRANAVDGLRSLLYLEEKTREHLQRQTPHVAVDELFAPPPASLCDGIEFLE   300
PA14_03000      FNDGFRAAPTRANAVDGLRSLLYLEEKTRERLQRQTPHVAVDELFAPPPASLCDGIEFLE   300
                                                       ** : :        **:**:*

HpdPAO1         FTAPDAKGIEQLRQLFNMMGFTETAKHRSKEVFLFQQNDINIVLNGSPTGHVHEFALKHG    82
HpdPA14         FTAPDAKGIEQLRQLFNMMGFTETAKHRSKEVFLFQQNDINIVLNGSPTGHVHEFALKHG    82
PA0242          FAVDETLGA-RLGQWLQRLGFARAGEHRSKNVSLLRQGDINLVLNAEPYSFAHGFFEAHG   359
PA14_03000      FAVDETLGA-RLGQWLQRLGFARAGEHRSKNVSLLRQGDINLVLNAEPYSFAHGFFEAHG   359
                *:. :: *  :* * :: :**:.:.:****:* *::* ***:***..* ...* *   **

HpdPAO1         PSACAMAFRVKNASQAAAYAESQGAKLVGSHANFGELNIPSLEGIGGSLLYLVDRYG-DR   141
HpdPA14         PSACAMAFRVKNASQAAAYAESQGAKLVGSHANFGELNIPSLEGIGGSLLYLVDRYG-DR   141
PA0242          PSLCATALCVRDAGQALERARAYGGQPYRGLLGPNEREIPAVRALDGSLLYLVERHTEGR   419
PA14_03000      PSLCATALRVRDAGQALGRARAYGGQPYRGLLGPNEREIPAVRALDGSLLYLVEQHTEGR   419
                ** ** *: *::*.**   *.: *.:   .     * :**::..: *******:::   *

HpdPAO1         SIYDVDFEFIEGRSANDNSVGLTYIDHLTHNVKRGQMDVWSGFYERIANFREIRYFDIEG   201
HpdPA14         SIYDVDFEFIEGRSANDNSVGLTYIDHLTHNVKRGQMDVWSGFYERIANFREIRYFDIEG   201
PA0242          SIYDSDFVTN---DADTSGLGLRRVDHVALALPAEGLDSWVLFYKSLFDFGADDEVVLPD   476
PA14_03000      SIYDSDFVTN---DADTSGLGLRRVDHVALALPAEGLDSWVLFYKSLFDFGADDEVVLPD   476
                **** **      .*: ..:**  :**::  :    :* *  **: : :*     . :

HpdPAO1         KLTGLFSRAMTAPCGKIRIPINESADDTSQIEEFIREYHGEGIQHIALTTDDIYATVRKL   261
HpdPA14         KLTGLFSRAMTAPCGKIRIPINESADDTSQIEEFIREYHGEGIQHIALTTDDIYATVRKL   261
PA0242          PYGLVTSRAVRSPCGSVRLPLNISEDRNTAIARSLSSYRGSGVHHIAFDCADIFAAVAQA   536
PA14_03000      PYGLVTSRAVRSPCGSVRLPLNISEDRNTAIARSLSSYRGSGVHHIAFDCADIFAAVAQA   536
                    : ***: :***.:*:*:* * * .: * . : .*:*.*::***:   **:*:* :

HpdPAO1         RDNGVKFMSTPDTYYEKVDTRVAGHGEPLEQLRELNLLIDGAPGDDGILLQIFTDTVIGP   321
HpdPA14         RANGVKFMSTPDTYYEKVDTRVAGHGEPLEQLRELNLLIDGAPGDDGILLQIFTDTVIGP   321
PA0242          KEAGVALLEIPLNYYDDLAARFDFDDEFLSELAYYNVLYDRD-AQGGELFHVFTEPFEER   595
PA14_03000      KEAGVALLEIPLNYYDDLAARFDFDDEFLSELAYYNVLYDRD-AQGGELFHVFTEPFEER   595
                :  ** ::. * .**:.: :*.  . * *.:*   *:* *   .: * *:::**: .

HpdPAO1         IFFEIIQRK-GNQGFGEGNFKALFESIEEDQIRRGVI----    357
HpdPA14         IFFEIIQRK-GNQGFGEGNFKALFESIEEDQIRRGVI----    357
PA0242          FFFEILQRRHGYAGYGAANVPVRLAAM--AQARRGVRRVKL    634
PA14_03000      FFFEILQRRHGYAGYGAANVPVRLAAM--AQARRGVRRVKL    634
                :****:**:  *  *:*  .*.  . : ::    * ****
```

B.

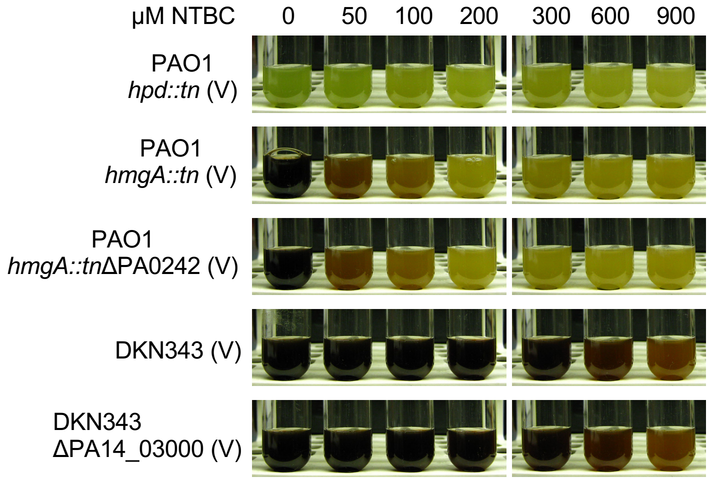

S4 Fig. PA0242/PA14_03000 is not involved in NTBC resistance. (A) Clustal O (1.2.0) sequence alignment of *P. aeruginosa* PAO1 and PA14 Hpd, PA0242 and PA14_03000 amino acid sequence. Hpd has sequence similarity to the C-terminal portion of the hypothetical proteins PA0242 and PA14_03000. The iron cofactor binding sites for Hpd (H168, H246, and E325) are highlighted in red. Asterisks indicate invariant amino acids; colon indicates conservation between groups of strongly similar properties; periods indicated conservation between groups of weakly similar properties. (B) Deletion of PA0242/PA14_03000 had no effect on NTBC sensitivity in the pyomelanin producers *hmgA::tn* and DKN343, respectively. *hpd::tn* was the non-pyomelanogenic control, while *hmgA::tn* was a positive control for pyomelanin production. Cultures were grown with the indicated concentrations of NTBC.
