# Supplementary material for: Characterization of 2-(2-nitro-4-trifluoromethylbenzoyl)-1,3-cyclohexanedione resistance in pyomelanogenic Pseudomonas aeruginosa DKN343
Source: PLoS One. 2017 Jun 1;12(6):e0178084. doi: 10.1371/journal.pone.0178084 (PMC5453437; doi:10.1371/journal.pone.0178084)
Supplement: S5 Fig — (A) EDTA treatment of cells increased outer membrane permeability, as measured by extracellular β-lactamase activity via nitrocefin hydrolysis. β-lactamase activity (U/L/OD600) for each untreated parent strain (0 mM EDTA) was set to 100% and percent β-lactamase activity was calculated following 0.1 mM EDTA treatment. Five biological replicates were tested. ANOVA followed by Tukey HSD post-hoc analysis: **, p<0.01; ***, p<0.001, n.s.; not significant. (B) hmgA::tn and DKN343 were treated with 0 or 0.1 mM EDTA and sub-inhibitory concentrations (0, 50, or 900 μM) of NTBC, as indicated, for 24 hours. hmgA::tn was the positive control for pyomelanin production. Three biological replicates were tested in triplicate. A representative data set is shown here. (PDF) [file pone.0178084.s005.pdf]

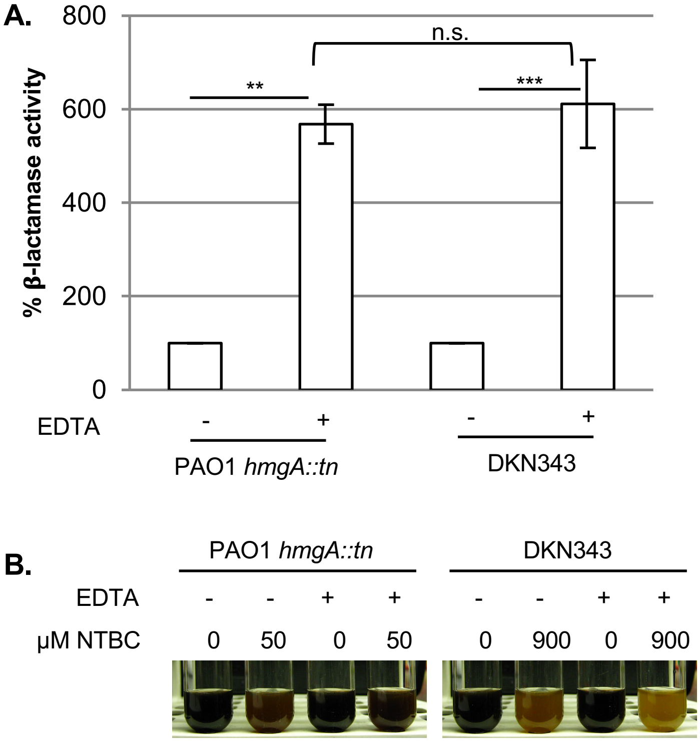

S5 Fig. An increase in outer membrane permeability does not result in increased sensitivity to NTBC in pyomelanogenic *P. aeruginosa*. A) EDTA treatment of cells increased outer membrane permeability, as measured by extracellular β-lactamase activity via nitrocefin hydrolysis. β-lactamase activity (U/L/OD_{600}) for each untreated parent strain (0 mM EDTA) was set to 100% and percent β-lactamase activity was calculated following 0.1 mM EDTA treatment. Five biological replicates were tested. ANOVA followed by Tukey HSD post-hoc analysis: **, p<0.01; ***, p<0.001, n.s.; not significant. **B)** *hmgA::tn* and DKN343 were treated with 0 or 0.1 mM EDTA and sub-inhibitory concentrations (0, 50, or 900 µM) of NTBC, as indicated, for 24 hours. *hmgA::tn* was the positive control for pyomelanin production. Three biological replicates were tested in triplicate. A representative data set is shown here.
