# Supplementary material for: Characterization of 2-(2-nitro-4-trifluoromethylbenzoyl)-1,3-cyclohexanedione resistance in pyomelanogenic Pseudomonas aeruginosa DKN343
Source: PLoS One. 2017 Jun 1;12(6):e0178084. doi: 10.1371/journal.pone.0178084 (PMC5453437; doi:10.1371/journal.pone.0178084)
Supplement: S1 Table — (DOCX) [file pone.0178084.s006.docx]

**S1 Table. List of primers used in this study.**

| **Primer name** | **Primer Sequence** (5’ – 3’)^a,b^ |
| --- | --- |
| **Primers for in-frame deletions** | |
| Δ*hpd*-up-For | GTAGCGGATCCCGATGCCTGCCACCGGAC |
| Δ*hpd*-up-Rev | GAGGCTGGCGGCAGCGGGACCGGCCTCCTCGTTGTTC |
| Δ*hpd*-dn-For | GAACAACGAGGAGGCCGGTCCCGCTGCCGCCAGCCTC |
| Δ*hpd*-dn-Rev | CGCCGAAGCTTGCCGCGGTGAAGCCGAGC |
| Δ*mexA*-up-For | CCTCTAGACATCACCGGCAACCTGACCC |
| Δ*mexA*-up-Rev | CCCCTTGATCAGCCCTTGCTTCGTTGCATAGCGTTGTCCTC |
| Δ*mexA*-dn-For | GAGGACAACGCTATGCAACGAAGCAAGGGCTGATCAAGGGG |
| Δ*mexA*-dn-Rev | GGGGTACCTACGGGTAGACCACCTTC |
| ΔPA0242-up-For | CCAAGAGCTCAACCACAGGCGGGTGGTC |
| ΔPA0242-up-Rev | GACAATTCTCACAGCTTGACACGCTGCATGCTTCGACTC |
| ΔPA0242-dn-For | GAGTCGAAGCATGCAGCGTGTCAAGCTGTGAGAATTGTC |
| ΔPA0242-dn-Rev | GTCCAAGCTTCCTGTGGGTCGCCGAGGTG |
| **Primers for gene sequencing** | |
| *hmgA*-external forward | CGATAAAAATAACGCAGCCAGC |
| *hmgA*-external reverse | GGTTTTGCAGGGGAAAGTCG |
| *hpd*-external forward | GGCATCTCCCATGTCGTCGGC |
| *hpd*-external reverse | GCCGCTGGAACGGAAACGC |
| **Primers for complementation** | |
| *hmgA*-for-pSB109 | GGCATATGATGAACCTCGACTCCACTGC |
| *hmgA*-rev | CCGAGCTCTTATCTCCGTTGCGGGTTG |
| *mexA*-for-pSB109 | CCCATATGATGCAACGAACGCCAGCC |
| *mexA*-rev | GGGAGCTCTCAGCCCTTGCTGTCGGTTTTC |
| PA0242-for-pSB109 | GGGAATTCATGCAGCGTTCGATCGCC |
| PA02424-rev | GGGAGCTCATTCTCACAGCTTGACCC |
| **Primers for site directed mutagenesis** | |
| *hmgA*_PAO1_-A306T-for | CAGCGTCCCCGGCCTG**ACC**AACATCGACTTCGTG |
| *hmgA*_PAO1_-A306T-rev | CACGAAGTCGATGTT**GGT**CAGGCCGGGGACGCTG |
| *hmgA*_PAO1_-H330Y-for | CCGTCCGCCATGGTTC**TAC**CGCAACCTGATGAACG |
| *hmgA*_PAO1_-H330Y-rev | CGTTCATCAGGTTGCG**GTA**GAACCATGGCGGACGG |
| *hmgA*_343_*-*T306A-for | GTCCATGGCCTG**GCC**AACATCGACTTC |
| *hmgA*_343_*-*T306A-rev | GAAGTCGATGTT**GGC**CAGGCCATGGAC |
| *hmgA*_343_*-*Y330H-for | CCGCCATGGTTC**CAC**CGCAACCTGATG |
| *hmgA*_343_*-*Y330H-rev | CATCAGGTTGCG**GTG**GAACCATGGCGG |

^a^Restriction enzyme sites are underlined.

^b^Mutagenic codons are in bold.
